# Evaluation of the function of the hippocampus at the preoperative stage of cardiac surgery as a harbinger of postoperative psychosis

**DOI:** 10.1192/j.eurpsy.2021.1949

**Published:** 2021-08-13

**Authors:** A. Sidenkova

**Affiliations:** Psychiatry, Ural State Medical University, Yekaterinburg, Russian Federation

**Keywords:** preoperative stage of cardiac surgery, postoperative psychosis, hippocampus

## Abstract

**Introduction:**

Development of an acute cerebral dysfunction in a form of delirium after cardiac surgeries is common general medical problem that associated with prolonged hospital stay after the surgery, risk of development of infection, risk of subsequent neurocognitive changes, and postoperative morbidity

**Objectives:**

To compare risk of development of postoperative delirium in elderly patients with and without hippocampal dysfunction

**Methods:**

Selective observational longitudinal study of the same group of objects in pre and postoperative period

**Results:**

For the diagnosis of degenerative process in CNS on early stages Free and cued selective reminding test immediate recall (FCSRT-IT) was shown to be the most sensitive. Based on learning of verbal material and semantic cues with recalling, FCSRT-IT allows differentiating amnestic disturbances hippocampal type from secondary disturbances of memory due to neurodynamic changes

**Conclusions:**

Hippocampal dysfunction is a factor of developing of postoperative delirium in elderly patients that requires using additional measures in patients with mild cognitive disturbance to prevent developing of postoperative delirium

**Disclosure:**

No significant relationships.

